# Bilateral Orchidopexy in a Hypochondriacal (Somatic Symptom Disorder) Patient and Determination of Fitness for Bilateral Orchidectomy

**DOI:** 10.1155/2016/8608951

**Published:** 2016-07-11

**Authors:** A. U. Nwaopara, Erefagha Leonardo P. Allagoa

**Affiliations:** Department of Mental Health, Federal Medical Center, Yenagoa, Bayelsa State 234, Nigeria

## Abstract

*Introduction.* DSM-5 identifies two disorders: somatic symptom disorder and illness anxiety disorder, to replace hypochondriasis in DSM-IV. Patients with both disorders are intensely anxious about the possibility of an undiagnosed illness or devote excessive time and energy to health concerns and are not easily reassured. Both disorders cause considerable distress and life disruption, even at moderate levels. However, hypochondriasis (DSM-IV) is an indication for neither orchidopexy nor orchidectomy. This is the rationale for this report which is the first of its kind to the best of available literature. This is an original case report of interest to a particular clinical specialty of mental health but it will have a broader clinical impact across medicine.* Case Presentation*. A 30-year-old black male presented to a primary care clinic with multiple internet searches on the topic of testicular pain and its differential diagnosis. He had a bilateral orchidopexy for a suspected torsion. He was referred to mental health unit, to determine fitness for further surgery.* Conclusions*. If hypochondriasis is suspected in a medical or surgical inpatient, a psychological medicine consultation should be performed, to elucidate the diagnosis, to avoid unnecessary procedures, and to optimize patient's care.

## 1. Introduction

Orchidopexy (or orchidopexy) is a surgery to move an undescended (cryptorchid) testicle into the scrotum and permanently fix it there. Orchidopexy also typically describes the surgery used to resolve testicular torsion [1]. Orchiectomy is the surgical removal of one or both testicles, or testes, in the human male. It is also called an orchidectomy, particularly in British publications [2].

Hypochondriasis as in the other somatoform disorders is among the most difficult and most complex psychiatric disorders to treat in the general medical setting. On the basis of many new developments in this field, diagnostic criteria have been revised to facilitate clinical care and research [3]. As with all psychiatric disorders, the somatoform disorders demand creative, rich biopsychosocial treatment planning by a team that includes primary care physicians, subspecialists, and mental health professionals [3]. This however is often taken for granted.

Hypochondriasis is now being directly or indirectly evaluated in numerous specialties, including cardiology, dermatology, otorhinolaryngology, gastroenterology, infectious disease, obstetrics and gynecology, ophthalmology, surgery, and oncology [4]. In this setting, the patient's report of his or her physical symptoms may not be specific, and the physical examination may not support a clear medical diagnosis, often leading to diagnostic puzzle [4, 5]. A history of “doctor shopping” is common. Because multiple evaluations and workups have been unfruitful, frustration may ensue, as the patient feels the physicians do not care, sometimes leading to mood problems like depression [5].

Some of the “tests” patients present are predictable. Often, the patient requests a specific blood test, radiological study, or invasive procedure or a combination thereof [6]. The patient may question the expertise of the primary physician and request referrals to different specialists and an ongoing workup. Any possible yield of an investigation must be balanced by the potential medical risks as well as the psychological risk of reinforcing the cyclical hypochondriacal pattern [6]. The physician should engage the patient in the decision-making process whenever possible.

The newly approved Diagnostic and Statistical Manual of Mental Disorders (DSM-5) contains many revisions, but few are as sweeping as those involving somatoform disorders. In the updated edition, hypochondriasis and several related conditions have been replaced by two new, empirically derived concepts: somatic symptom disorder and illness anxiety disorder [7, 8]. They differ markedly from the somatoform disorders in DSM-IV. Therefore to meet the criteria for somatic symptom disorder, patients must have one or more chronic somatic symptoms about which they are excessively concerned, preoccupied, or fearful. These fears and behaviors cause significant distress and dysfunction, and although patients may make frequent use of health care services, they are rarely reassured and often feel their medical care has been inadequate [7, 9]. Hypochondriasis is a highly disabling condition. Patients with hypochondriasis were found to view their health as worse than and to exhibit more severe psychiatric symptoms than age- and sex-matched control subjects [9].

Relatively little is known about the epidemiology of hypochondriasis, especially in the general population. The few population-based studies using interviews consistently reveal no or only very few hypochondriasis cases [10]. Only studies based on less restrictive inclusion criteria or studies using self-report questionnaires revealed higher case numbers. These prevalence rates varied from 4% to 10% in population-based samples [11].

Hypochondriasis has been investigated more often in medical patients. In general medical practice, the prevalence of hypochondriasis varied between 0.8% and 9% [12], and a median prevalence of 4.2% in seven primary care samples has been reported [13]. Most of these studies indicate that the hypochondriac concerns appear quite often in medical settings [12]. However, clinical samples may be influenced by selection bias or low index of suspicion [12, 13].

This report therefore aims to present the unusual presentation in a surgical setting and the avoidable but bizarre surgery done on a young male with clearly somatic symptom and illness anxiety disorders and the implication of these to medical practice, on the need for better collaboration between different specialties in medicine, in order to optimize patients care.

## 2. Case Presentation

A 34-year-old Nigerian man presented to the Mental Health/Psychiatry Clinic following a referral from the Surgical Outpatient Department on account of persistent testicular pain of a psychogenic nature and an insistence on a bilateral orchidectomy. History shows bilateral testicular pain of six-year duration, initially affecting the left testes alone, with the pain radiating to the perineum and pelvis, associated with pins and needles sensation and said to have affected his sleep and sex life. Although there is a positive history of multiple heterosexual partners, barrier contraception in the form of condoms was practiced. There was no history of fever and other constitutional features, undescended testes, childhood mumps, steroid Prune belly syndrome, perineal trauma, scrotal sores/or itching, exanthematous and pigmentary changes to the scrotum, change in testicular consistency, penile discharge, and pain on micturition and masturbation.

Testicular pain is, however, associated with inability to perform the sexual act as well as masturbation.

Due to the persistence of symptoms, he proceeded to General Hospital Ahoada, Rivers State, Nigeria, where a battery of unknown tests were carried out (involving drawing blood and several X-rays and ultrasound scans), results did not detect any abnormalities, drugs were prescribed, and he cannot recall their names; however, the pain persisted. Subsequently, he proceeded to a tertiary center (BMH, PH), where further tests were done and drugs were administered on an outpatient basis; however, the pain was persistent. He then proceeded to Navy Hospital, Borokiri, Port Harcourt, Nigeria, where an abdominal X-ray as well as abdominal and testicular ultrasound scans was done, as well as a thorough physical examination of the external genitalia; he was told that nothing was wrong with him and drugs were administered, but the symptoms were persistent. Similar findings were reported when he went to a tertiary health facility, UPTH. He also went to unorthodox, tradomedical practitioners in a quest for a permanent solution; several unknown concoctions were administered to him as well as incantations, all to no avail. He came to this facility in 2010 (after relocating to Bayelsa State, Nigeria); the battery of tests aforementioned earlier were made, including semen analysis (obtained via masturbation, a fact, he had earlier denied but is present in his medical record), and he began complaining a feeling that the right testis was ascending up into his stomach; a surgery (nature of surgery unknown to him) was performed in March, 2014; on recovery and discharge, pain persisted, with no worsening or relief; he claims that dribbling of urine began after the surgery.

There were associated feelings of worthlessness following the pain symptoms, loss of “manliness/sexual ability,” suicidal ideation, and suicidal attempts of 6-year duration, which has increased in intensity, frequency, and duration and progressively worsened after visits to doctors/several hospitals did not provide relief. Feeling of worthlessness was associated with low energy, loss of interest in pleasurable activities, cessation of self-employed work, loss of income, decreased willpower to live, loss of self-esteem, and feeling of extreme helplessness; however, patient claims he has a zest for life. Suicidal ideation is of 5-year duration; following failed attempts at obtaining a cure after consulting unorthodox medical practitioner, he began thinking about taking poisons to end his life; however, he did not tell anyone about his thoughts; there were no associated delusions, hallucinations, or other disturbances perceptions. Suicidal attempts began 4 years earlier; he ingested “poisonous” plants, battery acid, and “Baygon” insecticide. There is no history of attempts to end his life by violent means, self-mutilation, or ingestion of sleeping pills. Suicidal attempts were not revealed to anyone; however, the last “attempt” to end his life occurred 3 weeks prior to psychiatric consultation, with his mother privately reporting that he came to her privately, weeping bitterly and showed her some ropes he had in his possession for the purpose of killing himself, but he never carried through with the plan; he summarily denied this but later admitted it as a fact. Feeling of loss of “manliness” was of 5-year duration and began after he noticed that he could not have sex anymore due to the intense pain. This was associated with an increased desire to have his testicles removed, in order to be relieved of the pain. Although he considers himself as male, he insists he feels unmanly and incomplete; there was, however, a strong insistence that he is not a female on further probing during history taking.

However, no history of increased activity, agitation, restlessness, aimless wandering, increased libido, irrational talk, loss of appetite and sleep disturbances as well as hearing voices, social withdrawal, paucity of self-care, violent behavior, gathering of debris, or any abnormal behavior. Past psychiatric history revealed that there was a positive history of psychoactive drug use, specifically,* Cannabis*, which he used via smoking of dried leaves wrapped in a strip of paper; he began using it at age 16/17 (17 years ago); he was introduced to it by friends and a desire to experiment and rebel; he used drugs independently and with friends; habit was sustained by his allowance and stealing from his parents; drug use continued for a few years and ceased in his early twenties. In his mid-twenties, in 2006 (9 years ago), he became socially withdrawn, left the house for no apparent reason, and began living alone; then he disappeared for 3-4 months (he later admitted he travelled to Niger in a failed bid to emigrate to Europe) and returned home voluntarily, only to begin living in uncompleted buildings and talking irrationally, with history of provoked violent attack on a shopkeeper after being stigmatized as “a mental case.” Subsequently, in 2008, he was admitted to the Neuropsychiatric Hospital, Rumuigbo, and a diagnosis of “Substance Use Disorder” was made; several investigations were done and drugs administered; he was discharged after a month and a fortnight. Follow-up was regular for two months; however, he defaulted because he claimed he was not “mental” and because of the side effects of drugs. There is no history or evidence of a relapse following discharge. In the past medical history, there was no history of hypertension, epilepsy, asthma, diabetes mellitus, sickle cell disease, neurocutaneous disorders, and erectile dysfunction. Family history showed that he is the only male as well as the first of four children in a monogamous setting. However, his mother had 2 children in a later marriage after the divorce of his parents, making a total of 6 children, 3 full sisters and 2 half-sisters. His mother is a middle aged matron with tertiary level of education. His father is deceased, had tertiary level of education, and was a senior civil servant with the Ministry of Agriculture. He was not close to his father and appeared to have had a troubled relationship with him following the divorce of his parents; his father died three years earlier, at age 61, from an unknown medical condition. The personal history revealed that the patient completed his basic education. However, he did not pass the UME with good scores and failed to obtain a university admission, and he was said to be an average student. He enrolled in a part time program, in the National Open University, 2 years earlier, but did not complete the course because of his present illness. Subsequently, he said he has received training as an electrician and construction worker. He claims to be a single father of an 18-year-old boy (with elaborate information about the nonexistent child); however, his mother denies this and he later admitted he told us this so that we would remove his testes since he has a child. Social history showed that, in the past, he occasionally drank alcoholic beverages but has seldom done so for the past seven years. He smoked tobacco in cigarette form occasionally. Patient seldom interacts with people except his family members whom he lives with; he initially said he lives alone but his mother said he lives with her and he admitted this. Psychosexual history as elicited showed that adrenarche was attained at age 14 while onset of nocturnal emissions began at age 17. First sexual activity was with a female partner, experience was pleasurable and exciting, and subsequent experiences have been pleasurable and in almost all cases barrier contraception in the form of male condoms was used. The patient insisted that his sexual orientation and love interest has been strictly heterosexual. He has seldom had any early morning erections over the last 6 years and cannot recall having experienced any nocturnal emissions within the same period. He denies any masturbation as well as orgasm/ejaculation; however, semen for analysis was obtained via masturbation without the use of pornography. In the forensic assessment history, patient has not been convicted for any crime, sentenced to jail, or jumped bail; however, he has been arrested thrice and detained for minor offences such as moving at night during a curfew. Premorbidly, leisure activities included watching football matches; he formed deep ties in relationships, had few friends, and was introverted/quiet. His prevailing mood was happy.

### 2.1. Mental State Examination

Appearance and behavior showed a well dressed and well groomed young man (with attention paid to minute details such as manicured nails and perfectly styled hair) appearing appropriate for age, maintaining good eye contact, and holding an umbrella with depressed posturing. The tone of speech was low to moderate tone, reactive to circumstances, coherent, and relevant. The mood was sad and affect was depressed. Perceptual abnormalities present included somatic hallucination. There were no disorders of the thought process or formal thought disorder detected. Under cognition, patient was fully oriented to time, place, person, and circumstance. Attention, concentration, comprehension as well as calculator ability, and abstraction were within normal range. In addition, immediate recall, long and short memory are within normal range. Judgement was good and insight was partial (he did not believe the drugs will be of any benefit and his case is hopeless).

### 2.2. Diagnosis

Primary diagnosis was hypochondriasis with comorbid secondary diagnosis of severe depression with psychotic features.

### 2.3. Plan

Patient was managed using the biopsychosocial model. Drug treatment was done using tricyclic antidepressant (Amitriptyline) and antipsychotic medication (Olanzapine). Psychotherapy carried out involved cognitive behavioral therapy. Finally a letter was sent to SOPD about diagnosis and unsuitability for bilateral orchidectomy as requested.

### 2.4. Progress

Initial improvement (evidenced by reduction in pain intensity) was seen for the first 3 weeks; however, pain has been persistent. There was insistence on a bilateral orchidectomy during his last visit.

## 3. Discussion

The findings from the patient reviewed agree with the fact that hypochondriasis is evaluated directly or indirectly in numerous medical settings and is a diagnosis of exclusion [4]. In our patient and as in many cases previously described [3, 4, 14] the diagnosis was delayed because of the unusual clinical presentation. The clinical presentation is frequently confounding, especially in developed countries [4]. The result of this is that patient's report may not be specific and physical examination does not lead to a clear medical diagnosis, leading to diagnostic puzzle [4, 5].

- Although there are compelling pharmacologic data supporting the hypothesis that hypochondriasis is an obsessive-compulsive spectrum disorder, the comorbidity data are equally compelling in dispelling that hypothesis [15]. It is noteworthy that patients with OCD have obsessions that mimic hypochondriasis in that they are recurrent and persistent and symptoms experienced are intrusive, but these worries extend beyond concerns about illness (e.g., to fear about a door being unlocked) and are often accompanied by thematically related compulsive compensatory behavior (e.g., lock checking), which was not the case in this patient being reported [16].
- The patient took psychoactive substance (tobacco) occasionally. This is so because many hypochondriacs abuse drugs as a means of coping with anxiety, arising from the fear of having an undiagnosed illness which can be emotionally overwhelming [17]. Hypochondriacs may become enticed with the short-term relaxation that comes from substance abuse, which may lead to addiction, which ultimately will worsen the perception of the nonexistent illness [17].
- The findings in this report agree with the fact that mood disorder is the most comorbidity associated with hypochondriasis and in this patient a result of the hypochondriacal symptoms [5]. Patients with hypochondriasis usually request many investigations and procedures, which was exhibited by this patient [6]. Moreover, reassurance of patients usually remains unsuccessful [7]. The case was not different in our patient who insisted that his medical care has been inadequate [7, 9]. Consequently, they frequently request to be referred [6]. This then leads to “doctor shopping” or what is referred to as the “revolving door syndrome” [5]. He was treated in several hospitals, General Hospital Ahoada, Braithwaite Memorial Specialist Hospital, and University of Port Harcourt Teaching Hospital, before being referred to Federal Medical Center, Yenagoa.
- This agrees with the fact that there are usually visits to more than one physician, and in extreme cases, there are even multiple hospitalizations and possibly surgery [3, 18].
- The patient had no other somatic symptom disorders and the symptoms noted were of no secondary advantage for him, thereby differentiating it from body dysmorphic disorder, malingering, and factitious disorder [16].

The implication of these to medical practice is the need for better collaboration between different specialties in medicine following unusual presentations, presenting with diagnostic puzzle, in order to optimize patients care and in order to avoid unnecessary procedures on the patients.

## 4. Conclusions

This case confirms the atypical presentations and the diagnostic puzzles are associated with hypochondriasis.

## Abbreviations

FWACP: Fellow of the West African College of Physicians
MBBS: Bachelor of Medicine, Bachelor of Surgery
MSc: Masters in Science
IFPE: International Federation of Psychiatric Epidemiology
WACP: West African College of Physicians
BMSH: Braithwaite Memorial Specialist Hospital
UPTH: University of Port Harcourt Teaching Hospital
UME: University Matriculation Examination
OCD: Obsessive-compulsive disorder
AFMHA: African Foundation for Mental Health Advocacy
SOPD: Surgical Outpatient Department.

## Additional Points

Authors' information: (1) Dr. A. U. Nwaopara is a Fellow of the West African College of Physicians with specialist interest in consultation-liaison psychiatry and public health aspects of mental health. He is currently a consultant forensic psychiatrist and Head of Department of Mental Health at Federal Medical Center, Bayelsa State, Nigeria, a multispecialty treatment center, and is the Chairman of the Trustees of the African Foundation for Mental Health Advocacy (AFMHA). (2) Dr. Erefagha Leonardo P. Allagoa holds MBBS and MSc and is currently a resident doctor, undergoing specialist postgraduate training in psychiatry, in a multispecialty treatment center.
